# Combining Load–Close–Homogenize with Testing, Removal, and Rollover Strategies to Repopulate PRRSV Elimination Breeding Herds Using PRRSV-Positive Weaned Gilts

**DOI:** 10.3390/vetsci12101012

**Published:** 2025-10-20

**Authors:** Yulong Hu, Kangning Zhao, Guangqiang Wu, Haozhou Hong, Tian Xia, Zhicheng Liu, Yijuan Wang, Chunqing Sun, Chaosi Li, Zhendong Zhang, Jianfeng Zhang

**Affiliations:** 1 Boehringer Ingelheim Animal Health (Shanghai) Co., Ltd., Shanghai 200000, China; yulonghu1@boehringer-ingelheim.com (Y.H.); chunqing.sun@boehringer-ingelheim.com (C.S.); 2 MOE Joint International Research Laboratory of Animal Health and Food Safety, College of Veterinary Medicine, Nanjing Agricultural University, Nanjing 210000, China; longxi618@126.com; 3 Fujian Jinsheng Breeding Co., Ltd., Sanming 365000, China; guangqiangwu14@163.com; 4 Guangdong Tinoo’s Food, Qingyuan 511500, China; hhzmoshou2023@163.com; 5 Fujian YongCheng Breeding Technology Group Co., Ltd., Fuqing 350300, China; tiansummer1984@163.com; 6 Guangdong Province Key Laboratory of Livestock Disease Prevention, Institute of Animal Health, Guangdong Academy of Agricultural Sciences, Guangzhou 510640, China; rainman136@aliyun.com; 7 State Key Laboratory of Swine and Poultry Breeding Industry, Guangzhou 510640, China; 8 Zhangzhou Disease Prevention and Control Center, Zhangzhou 363000, China; aixuewang@163.com; 9 Jiangsu Co-Innovation Center for Prevention and Control of Important Animal Infectious Diseases and Zoonoses, College of Veterinary Medicine, Yangzhou University, Yangzhou 225009, China

**Keywords:** PRRSV elimination, test and removal, rollover, load–close–homogenize, live virus inoculation, weaned gilt

## Abstract

**Simple Summary:**

In this study, two PRRSV elimination strategies, LCH and T&R, were integrated. Systematic, step-by-step PRRSV elimination protocols were also developed and executed. They encompassed pre-evaluation of the PRRSV elimination capacity of new repopulation farm, assessment of PRRSV status and strain diversity of the two supplier farms, analysis of the natural infection dynamics of PRRSV within the population, assessment of parameters for LVI material preparation and implementation, and detailed programs for monitoring changes from homogenized infection to whole herd elimination.

**Abstract:**

This study aimed to evaluate the effectiveness of combining load–close–homogenize (LCH), test and removal (T&R), and rollover strategies for PRRSV elimination in breeding herds using PRRSV-positive weaned gilts. Here, a novel strategy was explored for PRRSV elimination from more than 1500 weaned gilts, and we documented the process from PRRSV natural infection to elimination at the herd level. With LCH implementation, the herd achieved PRRSV-positive stability within 8 months. Consequently, by rolling in self-breeding PRRSV-naive gilts to replace PRRSV-positive weaned sows batch by batch, the time from being positive stable to negative was 13 months. A PRRSV-positive farm intending to retain its genes in its repopulate farrow to become a finished breeding farm can initiate PRRSV elimination from its weaned gilts; this will result in the first farrowing batch of piglets aged 8–10 weeks becoming PRRSV-negative after 8 months of herd closure. This approach offers a viable pathway for genetic retention and PRRSV elimination in breeding farms.

## 1. Introduction

Porcine reproductive and respiratory syndrome (PRRS), one of the most critical viral swine diseases, is caused by PRRS virus (PRRSV), an enveloped, positive-sense, and single-stranded RNA virus [1]. According to the most recent classification, the previous PRRSV genotypes have been classified into two species: Betaarterivirus suid 1 and Betaarterivirus suid 2 within the subgenera Eurpobartevirus and Ampobartevirus. Herein, we have used the commonly accepted, recognized conventional names PRRSV-1 and PRRSV-2 to denote the two PRRSV species [2]. PRRSV infection characterized by clinical symptoms such as reproductive disorders in pregnant sows and respiratory diseases in piglets poses a major threat to the global pig industry [3,4].

PRRSV infection has been reported in >30 countries throughout North America, Europe, Asia, and South America. Major outbreaks have been concentrated in regions with intensive pig production systems, such as the United States and Vietnam [5]. In a 2022 epidemiologic analysis of PRRSV infection at 100 intensive pig farms in 21 provinces of China, 73.6% (1780/2416) of the samples were positive for wild-type PRRSV, regardless of the sample type. Moreover, 58.1% (254/437) of the farm batches were positive for wild-type PRRSV, indicating a high prevalence of PRRSV infection across different regions in China. On the basis of the time of infection, 58.9% of the suckling piglets [processing fluid (PF)] and 30.8% of the weaning piglets (weaning serum) had early-stage PRRSV infection (at approximately 90% of the farms). Moreover, the sequencing analysis results suggested the presence of a wide variety of wild-type PRRSV strains in China, with lineage 1 being the dominant strain [6].

PRRSV affects the reproductive performance of sow herds and the production efficiency of growing herds. Over 2019–2021, PRRSV outbreaks led to a median total production loss (TL) of 3675 pigs per 1000 sows among breeding herds in the United States [7]. From the weaned to market stage, PRRSV unstable herds were noted to demonstrate a 3.3% increase in mortality, a 0.04% increase in feed conversion rate (FCR), and a 17.7 g decrease in average daily weight gain (ADWG) [8]. Compared with PRRSV stable herds, PRRSV elimination significantly increased live birth rate by 4.9% but reduced stillbirth rate and preweaning mortality by 3.0% and 8.8%, respectively [9].

The economic impact of PRRSV was estimated on the basis of production parameters. The total annual economic impact of PRRSV on US swine producers has been estimated to be $66.75 million in breeding herds and $493.57 million in growing pig populations [8]. A recent study in China indicated that the economic loss from outbreaks to regain the basic performance is ¥822.75 per sow, whereas the mean cost is ¥601.62 per sow in fattening herds (including nursery pigs); the overall economic impact of PRRSV on the entire herd is ¥1424.37 per sow [10]. Moreover, in the Chinese market, the 55-kg crossbred, PRRSV-naïve gilts have a premium of more than ¥200.

Several cases of PRRSV elimination in breeding farms have been reported thus far [11]. In October 2024, >40% of the farms in the Morrison Swine Health Monitoring Project were at status 4 [12]. However, the sites remained at status 4 for a median of 2 years, and the overall PRRS incidence rate after the sites achieved status 4 was 23.43 outbreaks per 100 farm years [13]. Moreover, the minimum duration of status 4 maintenance (to ensure the return on investment for PRRSV elimination) was 2.1 years [14]. This indicates the need for a pre-elimination evaluation to ensure that the farm can minimize the frequency of PRRSV introduction.

Three PRRSV elimination strategies are mainly employed in the field: (1) whole-herd depopulation and repopulation, (2) test and removal (T&R), and (3) load–close–homogenize (LCH) [15]. The depopulation strategy demonstrates the highest efficiency and lowest risk; however, the cost is excessively high for most pig herds [16]. It is typically implemented after a highly virulent PRRSV infection and concurrently involves the use of an offsite breeding farm to premate PRRSV-naïve gilts for repopulation. The T&R strategy is frequently used only in small-scale farms because it involves the use of expensive quantitative polymerase chain reaction (qPCR) tests and has a relatively low success rate [17]. LCH appears to be the only relatively inexpensive method and has thus emerged as the most preferred PRRSV elimination strategy. This method generally encompasses three steps: (1) introducing as many gilts as possible, (2) implementing herd closure until the herd attains status 2, and (3) exposing all animals to live PRRSV simultaneously [18].

Both live virus inoculation (LVI) and modified live vaccine (MLV) can homogenize herd immunity by synchronizing viral exposure or vaccine-induced immunity across the entire population; this can reduce viral circulation and accelerate PRRSV stabilization within a herd closure [19]. Applying LVI as a whole-herd exposure program for PRRSV control and elimination can shorten time to stability (TTS) compared with MLV application. In contrast, compared with LVI, MLV may reduce time to baseline productivity and TL from the outbreak setup to the recovery and productive baseline [20]. Comprehending the expectations of the key decision-makers throughout the PRRSV elimination period is the initial step. These expectations can aid in selecting between LVI and MLV as the immunization tool. LVI should be adopted if the decision-makers require the PRRSV-negative state to be attained as soon as possible, regardless of the cost. In contrast, if the decision-makers aim to minimize economic loss when successfully eliminating PRRSV, MLV immunization may be selected.

The current study was initiated to fulfill a request from a breeding group. The group repopulated a PRRSV-negative herd by introducing animals from the one of two PRRSV-positive farms to maintain genetic integrity and reduce costs. The farm owner aimed to sell the 50–55-kg PRRSV-negative gilts as soon as possible. Thus, a novel PRRSV elimination strategy was phased in, starting from stabilizing the herd through LCH to accelerating the birth of PRRSV-naïve individuals through T&R and rollover implementation. To our knowledge, this is the first study and elimination case to pre-evaluate the capacity of a PRRSV elimination farm, test and screen the supplier farms, and use LVI for homogeneous immunization to replace PRRSV-positive weaned sows for rollover. At the end of the study, we efficiently eliminated PRRSV from weaning gilts and recorded a large amount of PRRSV infection dynamic parameters throughout the entire process. The current results may facilitate the development and implementation of future PRRSV elimination projects.

## 2. Materials and Methods

### 2.1. Sample Collection and Laboratory Tests

Throughout the study period, all clinical samples were collected by each participating farm’s contract staff. All ready-to-test samples were stored at −20 °C until delivery to our laboratory within 48 h. Commercial tools for population-based sample collection of oral fluid (OF), tongue tip fluid (TTF), and PF were purchased from Harbin Pets Biotechnology (Harbin, China). Enzyme-linked immunosorbent assay (ELISA), polymerase chain reaction (PCR), and open reading frame (ORF) 5 sequencing were performed at Guangdong Academy of Agricultural Sciences. The official test reports were returned to the farms within 1 week after sample receipt. Commercial kits including HERDCHEK* PRRS X2 (IDEXX Laboratories, Westbrook, ME, USA) and VetMAX PRRSV EU & NA 2.0 (Thermo Fisher Scientific, Waltham, MA, USA) were used for serological and viral detection of PRRSV, respectively. PCR-positive samples with Ct values of <30 were randomly selected per batch and sent to Sangon Biotechnology (Shanghai, China) for ORF5 sequencing. IDEXX RealPCR CSFV RNA Mix (IDEXX Laboratories), IDEXX RealPCR ASFV Mix (IDEXX Laboratories), and PRV gE Real PCR Mix (GuanMu Diagnosis, Changsha, China) were used for qPCR, according to manufacturers’ instructions.

The phylogenetic tree construction and genetic similarity analysis were performed using megalign (DNAstar, Madison, WI, USA). The reference ORF5 sequences of lineage 1, 3, 5, and 8 of PRRSV-2 were obtained from the NCBI website. The Genbank codes and ORF5 sequences obtained in this study are listed in Supplementary Table S1.

We used the AASV standard to classify the PRRSV breeding herd statuses, with a slight modification to the criteria for status 4. Specifically, following the replacement of seven consecutive batches of sows with PRRSV-naïve gilts, 60 samples were randomly selected from the breeding herd. These samples were then tested by ELISA 30 days and 90 days later. The breeding herd was classified as status 4 if all the samples tested ELISA-negative [21].

### 2.2. Basic Information, Roadmap, and Timeline of Our Strategy for PRRSV Elimination from Weaning Gilts

Basic information on repopulation and supplier farms.

The repopulation farm JS has an inventory of 2400 sows. The aim was to repopulate the PRRSV elimination herd from two supplier farms SG and FK. All three farms belonged to one swine rearing group and were located in the same district.

JS operated on a three-weekly batch farrowing system from farrow to finish, thus involving 21-day intervals within batches. They did not have an air filter system set up. Its farms were separated into boar-stud, sow-herd, and wean-to-finish areas. The farm sold commercial parental breeding pigs to downflow farms and hogs to slaughterhouses.

SG and FK had an inventory of nearly 800 sows and operated on a 7-batch rearing system from farrow to finish. The farms provided 700 weaned gilts every 21 days. Therefore, with one source introducing weaned gilts, JS was completely filled within three batches.

### 2.3. Roadmap and Timeline for PRRSV Elimination from Weaning Gilts

**Figure 1:**
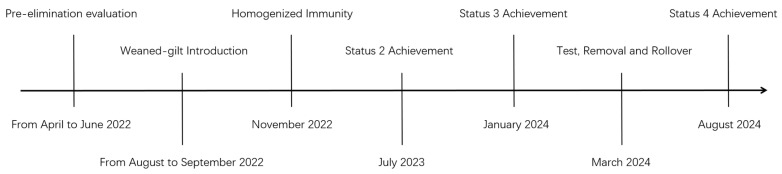
Roadmap and timeline for PRRSV elimination in the repopulation farm.

From April to June 2022, we performed pre-evaluation to JS for PRRSV elimination; the pre-evaluation criteria included proximity, conducive local climate, and appropriate facilities (to maintain PRRSV-naïve status for as long as possible). To shorten the PRRSV unstable 1-to-2 period, gilts were to be sourced from a single farm. Therefore, the PRRSV status and strains diversity were classified and evaluated in SG and FK farms. After pre-evaluation, >1500 weaned gilts were introduced from three consecutive batches from SG in August and September 2022. The herd was closed after the introductions were complete. After 8 weeks of quarantine, homogenized immunity was implemented in November 2022. Until the farrowing of Batch 1 gilts, the herd was verified to be PRRSV stable 2 in July 2023. After confirming the breeding herd was in status 2, we introduced 30 sentinel PRRSV-naïve gilts from Batch 1 into the sow herd. Three rounds of serological tests were performed; in January 2024, negative results were obtained in all three rounds, indicating that the herd had achieved status 3. When Batch 1 self-breeding gilts became 8 months of age, we initiated T&R and rollover to replace the antibody-positive weaned sows in March 2024. After antibody-positive sows in all seven batches were replaced by PRRSV-naïve gilts, 60 sows were randomly selected for ELISA testing. Following two rounds of ELISA testing, the results were proven to be negative in August 2024 (Figure 1). Subsequent section elaborates on each stage of our timeline and monitoring strategies.

### 2.4. Pre-Elimination Evaluation

The breakeven years of maintaining PRRSV status 4 influence the costs and returns of PRRSV elimination. Therefore, we performed pre-evaluation before elimination as the first decision-making step. Specifically, we evaluated the risk factors for PRRSV introduction: proximity, environment, and climate.

Proximity analysis was conducted by using Google Maps and farm visits; we assessed several factors, including the number of farms within 3 km of JS, the PRRSV status of the farms, their distances from the nearest public roads and villages, and the terrain surrounding them [22].

Weather information throughout 2021 in JS’s district was collected from the 2345 weather forecast website, including the number of months with temperatures <4 and <10 °C, annual mean wind speed and temperature, and the number of fog and haze days [23].

### 2.5. Evaluation of PRRSV Status and Genetic Diversity in Supplier Farms and Two Weaned Gilt Batches

Reducing PRRSV infection pressure minimizes weaning piglet mortality in the nursery phase [24]. PRRSV monitoring was implemented in SG and FK, as described in Table 1. In brief, three consecutive batches were used for monitoring, according to the AASV PRRSV herd classification [21]. We collected three types of samples to trace PRRSV circulation in the herd. First, PF, a population-based sample, was collected from all litters in a batch, and castrated tissues from every 30–50 L were pooled into one sample. Thirty serum samples were collected to detect at least 10% prevalence at weaning (at 21–25 days of age) [25]. Ingelvac PRRS MLV (Boehringer Ingelheim (Jiangsu) Animal Health, Taizhou, China) was used to vaccinate when the weaners arrived at the nursery barns. After 3 weeks, six OF samples were randomly taken for testing.

To reduce potential PRRSV strain diversity and shorten the unstable-to-stable period, positive samples were assessed through ORF5 sequencing [26]. From each batch at the two supplier farms, one or two positive samples with Ct values of <30 were randomly selected for ORF5 sequencing. Final, phylogenetic trees were prepared to analyze the lineage and number of strains circulating in the farms.

### 2.6. Monitoring PRRSV Infection Dynamics in Two of Three Weaning Gilt Batches

Three consecutive batches were introduced to JS and accommodated in five separate barns: Barns A and B for Batch 1, Barn C for Batch 2, and Barns D and E for Batch 3. The rearing area was set from 0.44 to 0.5 m^2^ so as to reduce the motility in the nursery period. At 11–12 weeks of age, gilts were transferred to finishing rooms batch by batch and accommodated into nine separate barns.

To monitor PRRSV infection rate and understand PRRSV infection dynamics further without any vaccination and cross-pen movement before mass homogenized infection, we monitored the PRRSV status of Batches 1 and 2 every 2 weeks after the arrival of weaned gilts to JS. Batch 3 was not monitored to limit costs. The surveillance was terminated only after all pens tested PRRSV-positive in qPCR testing, indicating that most gilts were naturally infected. The OF samples were collected using a rope with ≤20 pigs included per rope. The samples were collected for 20–30 min to ensure that as many gilts as possible chewed the rope [27]. Pens with low Ct values were marked to trace piglets with high viremia and used for LVI material preparation.

### 2.7. LVI Material Preparation and Homogenized Infection

**Figure 2:**
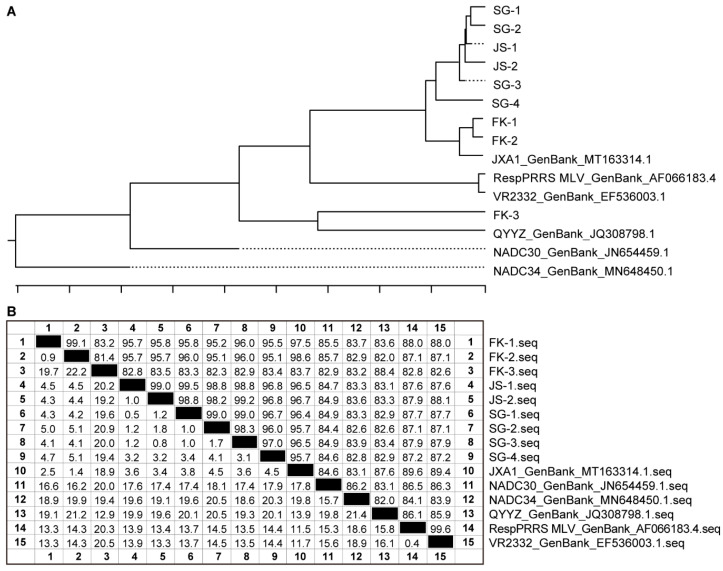
Phylogenetic tree and genetic similarity analysis based on ORF5 sequences. (**A**) Phylogenetic tree consolidated according to the farms SG, FK, and JS. (**B**) ORF5-based genetic similarity.

To achieve PRRSV herd status 2 as early as possible, the farm owner decided to use LVI for homogenized infection [20]. One PRRSV viremic gilt was screened according to the method mentioned in the previous section, 1 week before LVI. The maximum amount of blood was collected before euthanasia. The collected blood was centrifuged to obtain serum, which was then subjected to qPCR tests for African swine fever virus, classical swine fever virus, pseudorabies virus, and PRRSV. The serum sample was positive for PRRSV with a Ct value of 27.5; in contrast, it was negative for the other pathogens. Next, the sample was subjected to ORF5 sequencing, and the obtained sequence was compared with the ORF5 sequences from SG and the introduced batches (Figure 2); the results verified that PRRSVs were of the same lineage. However, virus isolation from serum and tissue samples was unsuccessful.

In PRRSV-positive populations, the immunization dose required to boost infection with the same PRRSV strain is lower [28]. Therefore, we diluted the serum seed 100-fold as the boost acclimation material; 3000 mL of the diluent was prepared by adding 1 g of ceftiofur per 250 mL of sterile saline [28]. Before use, the diluent was precooled on ice and used immediately after dilution to ensure virus activity maintenance.

To conserve the LVI materials and ensure a high infection success rate, LVI was performed intramuscularly. To minimize mortality due to LVI, active infection was performed in the gilt between 10 and 16 weeks of age.

One day before infection, the entire herd was administered enrofloxacin (Elanco Shanghai Animal Health, Shanghai, China). At 3 days after infection, the entire herd was treated with tilmicosin and tetracycline (Elanco Shanghai Animal Health) for 2 weeks.

### 2.8. Monitoring Strategy from LVI to PRRSV Status 2 Achievement

After mass LVI, we set up the monitoring schemes with three objectives: (1) ensuring active infection was successful, (2) evaluating infection rate to ensure the virus stops circulating within the herd, and (3) deciphering timepoints for shedding termination. The detailed method is described in Table 2. OF samples across all pens were collected after 12 to 24 h of active infection, and qPCR was used to evaluate infection efficiency. Shedding status was monitored every 4 weeks until all pens became qPCR negative.

To assess if the infection rate was >90% at 4 weeks after LVI, we randomly collected ≥30 serum samples and subjected them to ELISA. Next, exposure status was monitored until the end of T&R to trace the long-term changes in the sample/positive (S/P) value and roll over positive sows.

We used the sampling strategy described in Table 3 to ensure that the herd’s status 2 achievement. Four sample types were collected for evaluation: (1) TTF samples were collected from stillborn piglets to evaluate vertical transmission. In each sample, we included 20–100 tongue tips, as indicated previously [29]. (2) PF samples were collected to evaluate the early infection in suckling piglets using the aggregated method, described in the “Evaluation of PRRSV status and genetic diversity in suppliers SG and FK” subsection. (3) Serum samples (*n* ≥ 120) were collected from weaning piglets to evaluate the PRRSV status over the due-to-wean period and confirm that prevalence was <3% [25]. (4) Serum samples (*n* = 60) were collected from perinatal sows for qPCR analysis; serums samples from every five sows were pooled for analysis. The samples were evaluated until four consecutive testing results were negative, indicating that the herd had achieved status 2.

### 2.9. Monitoring Strategy from PRRSV Herd Status 2 to Status 4 Achievement

When the herd achieved status 2, the farm began weaning the gilts on site. In piglets, maternal antibodies typically disappear around 8 weeks of age [30]. To ensure that the self-breeding gilts could be used as PRRS naïve sentinel pigs for verifying whether the breeding herd had attained PRRSV status 3, we randomly selected 120 self-breeding gilts (age ≈ 8 weeks) from each batch to ensure that the positivity rate of ELISA did not exceed 3% (Table 4).

The first batch of 30 gilts was introduced to the gestation barn and allowed to commingle with the present sows; ELISA testing was performed before introduction and then at 30 and 60 days after introduction. If all samples were negative, the herd was confirmed to have achieved status 3, according to the AASV classification [21]. When the self-breeding gilts in the first batch became 8 months of age, the T&R and rollover protocol was implemented. Serum was collected from the sows at weaning and subjected to ELISA; positive sows were immediately culled and replaced with PRRSV-naïve gilts. This procedure was performed until all positive sows in all seven batches were rolled over (Table 4).

At 30 and 90 days after the completion of T&R and rollover, blood samples were collected from 60 randomly selected sows and subjected to ELISA. All samples were negative, and the herd was confirmed to have achieved PRRSV herd status 4 according to AASV classification [21].

### 2.10. Descriptive Statistics

Basic statistical indicators, including mean, coefficient of variation (CV), and positivity rate, were assessed using GraphPad Prism (version 10; GraphPad, La Jolla, CA, USA) and Excel 2023 (Microsoft, Redmond, WA, USA).

Fisher’s exact test for multiple independent samples was employed to evaluate the overall differences in positive rates among the 14 WPC groups (presented in Section 3.4) and 7 batches (shown in Section 3.6). It was executed using SPSS 26.0 (IBM Corp., Armonk, NY, USA), with a pre-defined significance level (α) set at 0.05. The null hypothesis (H_0_) postulated that the positive rates were identical across all groups or batches. Conversely, the alternative hypothesis (H_1_) posited that at least one group or batch exhibited a significantly different positive rate from the others.

Upon obtaining a significant overall test result (*p* < 0.05), pairwise comparisons of positive rates were carried out between all pairs of WPC groups and batches to pinpoint specific group differences. Considering the substantial number of pairwise comparisons, the Bonferroni correction was applied to control for Type I errors (false positives). For 14 WPC groups analysis in result 3.4, the corrected significance level (α’) was adjusted to 0.05/91 ≈ 0.00055, and for 7 batches analysis in result 3.6, it was set to 0.0024. A pairwise comparison was deemed statistically significant only when its *p*-value was less than 0.00055 and 0.0024 for 14 WPC groups and 7 batches respectively.

In the presentation of results, positive rates were arranged in descending order, and letter-based significance labeling was carried out in accordance with standard academic norms. Specifically: (1) the group with the highest positive rate was designated the letter “a”; (2) each subsequent group was compared with all preceding groups. If no significant difference was identified, it was assigned the same letter(s) as the non-significant group(s); (3) when a significant difference was detected, a new sequential letter (such as “b”, “c”, “d”, etc.) was assigned. Groups sharing the same letter imply no statistically significant difference in positive rates, whereas groups with different letters suggest significant differences at the corrected α′ level.

## 3. Results

### 3.1. Pre-Elimination Evaluation at JS

JS is located in a hilly area approximately 200 m above sea level and surrounded by hills on all sides. The pig farm is surrounded by banyan trees that are higher than the farm in all directions, except the southern side. The northern region is slightly elevated compared with the southern region. Two other pig farms are located within a 5-km radius of JS. One of them is located in the northeast direction of JS within a 3-km radius (actual distance = 2.78 km), and it has hills approximately 300 m above sea level in the middle. At the time of evaluation, neighboring farm was PRRSV-positive but unstable, and its size was approximately 300 heads. JS is approximately 1 km from a highway, about 200 m above sea level. JS has a 5–10-m-wide green belt in the middle for shielding, and its exhaust is directed toward the highway. As of 2022, the mean high and low temperatures in JS’s district were 27 and 16 °C, respectively, and the mean wind speed could be categorized as class 1, constituting a soft wind. In JS, the minimum temperature is <10 °C in only 3 months annually, and the temperature never decreases to <4 °C throughout the year. The area does not encounter any haze or sandstorm days throughout the year.

### 3.2. PRRSV Status Evaluation and ORF 5 Sequencing Analysis at SG and FK

Based on the PRRSV monitoring results, both supply farms exhibited a PRRSV herd positive unstable status (1). Among them, SG had a PCR positivity rate of <25% in the last monitoring batch, with the circulating isolates in all batches being lineage 8 (Figure 2A) and showing relatively low strain diversity. FK was classified to have an unstable high prevalence (1-A) status during the monitoring period, with the resident isolates including lineages 8 and 3 (Figure 2A). On the basis of the PRRSV positivity rate before weaning and the PRRSV genetic diversity, we decided to introduce three consecutive batches of weaning gilts from SG in a single source manner. Detailed PCR results are shown in Table 5 and Table 6, whereas the gene similarity information is illustrated in Figure 2B.

### 3.3. PRRSV Infection Dynamics in Two Weaning Gilt Batches Introduced from SS

**Figure 3:**
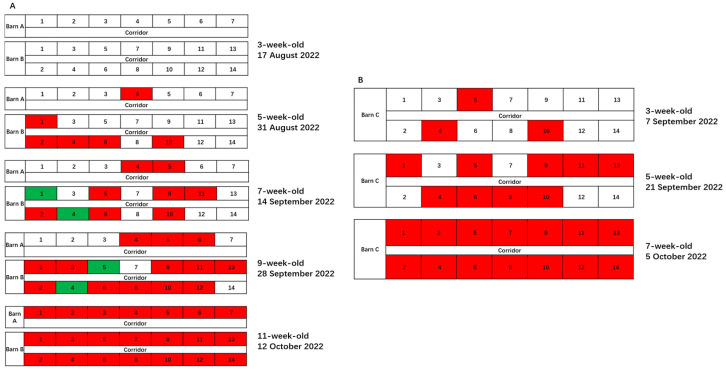
Dashboard of natural transmission of PRRSV within two batches after introduction. (**A**) Whole-herd qPCR used to continuously monitor Batch 1 at 2 weeks apart. (**B**) Whole-herd qPCR used to continuously monitor Batch 2 at 2 weeks apart. Red boxes indicate positive results of qPCR; Green boxes indicate qPCR results turning from positive to negative in the final two weeks.

Batch 1 of weaned gilts was introduced to JS on 17 August 2022. OF samples were collected from the first two of three batches on the day of arrival, and first batch was verified to be PRRSV-negative on PCR. Two weeks after introduction, six pens became PRRSV-positive on PCR, with a positivity rate of 28.6% (6/21). When the pigs were 11 weeks old (at 8 weeks after introduction), all pens became PRRSV-positive on PCR. On the day of Batch 2 accommodation, three pens were verified to be PRRSV-positive on PCR, with a positivity rate of 21.4% (3/14). When the pigs were 7 weeks old (at 4 weeks after accommodation), all pens were PRRSV-positive on PCR (Figure 3).

### 3.4. Homogenized Infection and Herd PRRSV Shedding Status Evaluation

**Figure 4:**
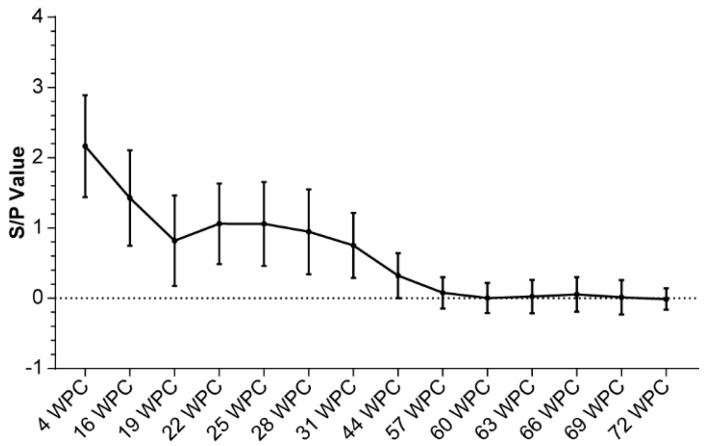
S/P values of sows over 4–72 post challenge weeks. Data are presented as means and standard deviations.

At 12–24 h after homogenized infection, all OF samples across the pens were PRRSV-positive on PCR, thereby validating the success of LVI. At 4 weeks after homogenized infection, 366 of 369 serum samples from sows were PRRSV N protein antibody-positive, indicating a 99% positivity rate (Table 7 and Figure 4).

**Figure 5:**
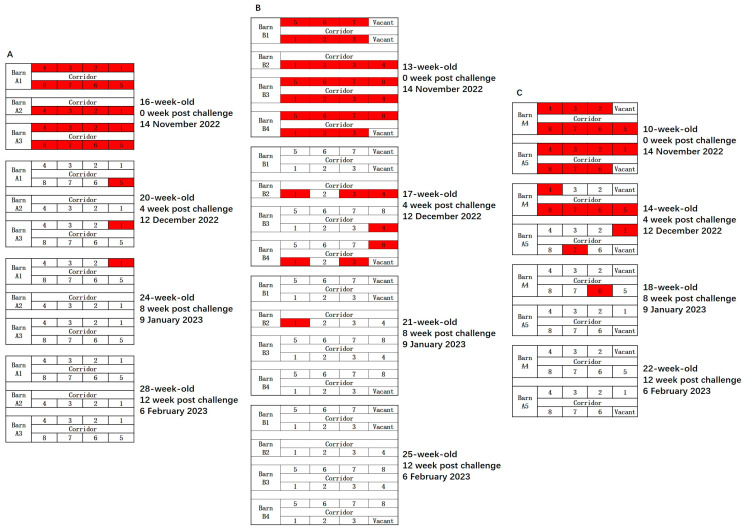
Dashboard of PRRSV diminishing from the herd in three batches after homogenized infection challenge. (**A**) Whole-herd qPCR used to continuously monitor Batch 1 at 4 weeks apart. (**B**) Whole-herd qPCR used to continuously monitor Batch 2 at 4 weeks apart. (**C**) Whole-herd qPCR used to continuously monitor Batch 3 at 4 weeks apart. Red boxes indicate positive results of qPCR.

LVI was performed in the Batch 1, 2, and 3 herds at the ages of 16, 13, and 10 weeks, respectively. For Batch 1, only two pens remained PRRSV-positive on PCR 4 weeks after infection, whereas the remaining 18 pens became negative. For Batch 2, seven pens were PRRSV-negative on PCR 4 weeks after infection, whereas 18 pens changed from PRRSV positive to negative. For Batch 3, 50% of the pens became PRRSV-negative on PCR 4 weeks after infection, indicating a 50% positivity rate. Finally, all OF samples from all three batches became PRRSV-negative on PCR 12 weeks after active infection (Figure 5).

### 3.5. Achievement of PRRSV Stable State in Breeding Herd

**Figure 6:**
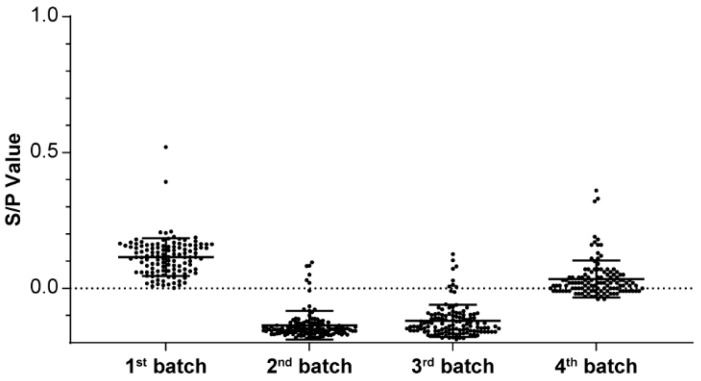
S/P values in 8-week-old piglets over four consecutive batches. Data are presented as means and standard deviations.

When Batch 1 herd began farrowing, we performed PRRSV monitoring through PCR testing of TTF, PF, and perinatal sow and weaning piglet serum samples; this was continuously implemented over four batches. The results demonstrated that all four sample types from Batch 1 herd were PRRSV-negative (Table 8 and Table 9), indicating that the herd had attained the positive stable 2 status in the first farrowing batch. To further verify the PRRSV exposure status of the self-breeding gilts, ELISA was performed in 8-week-old self-breeding gilts over four consecutive batches. The results revealed that all batches except Batch 1 were PRRSV-negative (Figure 6). The mean S/P values of the four batches were 0.11, −0.14, −0.12, and 0.03, respectively (Table 10).

### 3.6. Breeding Herd Status 4 Achievement After T&R and Rollover

**Figure 7:**
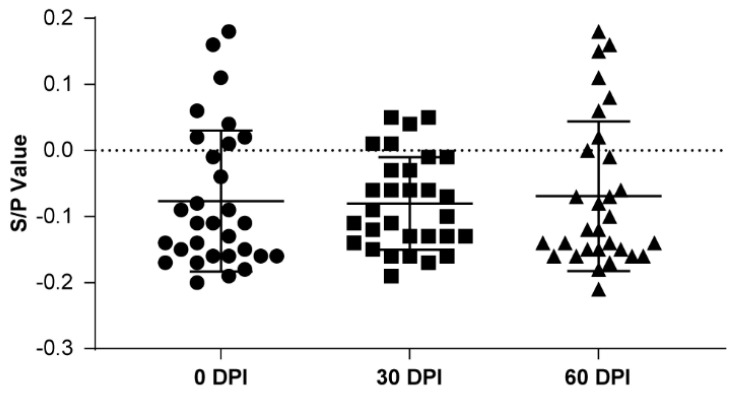
S/P values in 30 PRRSV-naïve sentinel gilts over 0–60 post introduction days. Data are presented as means and standard deviations.

**Figure 8:**
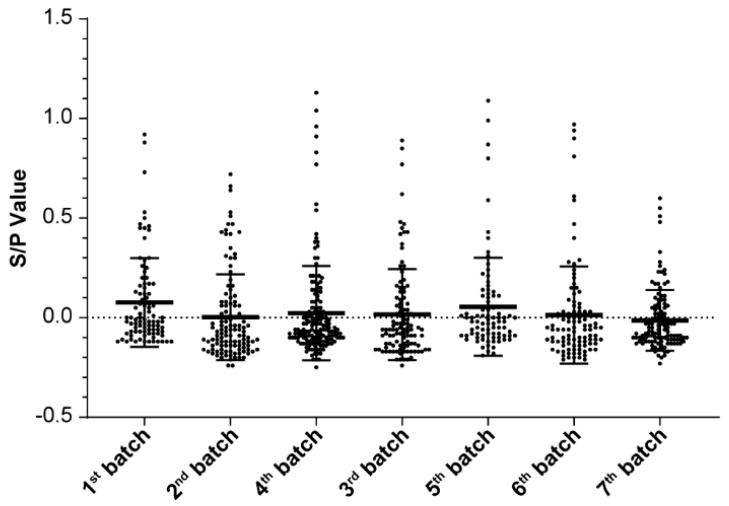
ELISA results of weaned sows in all seven consecutive batches.

**Figure 9:**
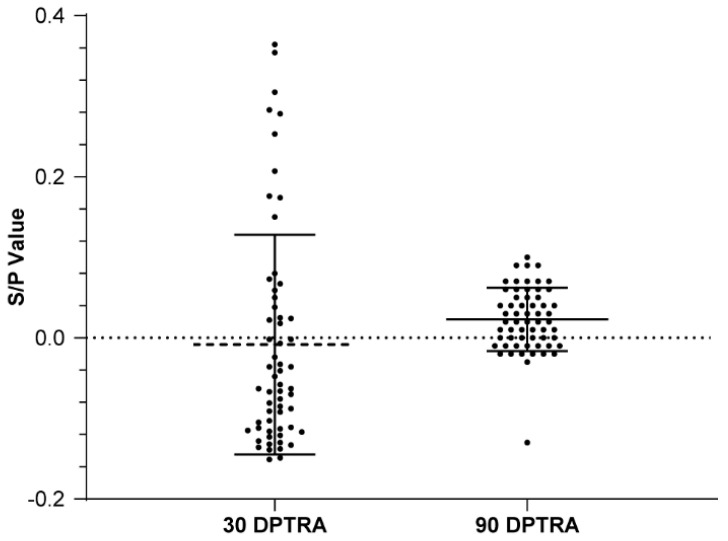
Results from two rounds of ELISA of 60 sows on 30 and 90 post-T&R days.

Before, 30 days after, and 60 days after 30 PRRSV-naïve self-breeding sentinel gilts were introduced, all serum samples were PRRSV-negative on ELISA (Figure 7), with mean S/P values of −0.08, −0.08, and −0.07, respectively, and CVs of 0.11, 0.07, and 0.11, respectively (Table 11). When self-breeding gilt Batch 1 became of mating age, ELISA testing was performed continuously on seven weaned sows batches (Figure 8); over time, the PRRSV positivity rate decreased from 12.2% to 3.3%, and the S/P value decreased from 0.077 to −0.013 (Table 12). At 30 and 90 days after implementing T&R and rollover in all seven consecutive batches, we randomly selected 60 samples per timepoint from the sow herd; all the samples tested PRRSV-negative on ELISA. At 30 and 90 days after T&R and rollover implementation, mean S/P values were −0.01 and 0.02, respectively, and CVs were 0.14 and 0.04, respectively (Table 13 and Figure 9). From then on, in the all following batches of the gilts and boars were all ELISA and PCR negative.

## 4. Discussion

PRRSV elimination approaches are relatively well-established, with numerous successful cases being reported [18,31,32]. Nevertheless, maintaining a PRRSV elimination status is rather challenging [33]. The mean duration of the PRRSV status 4 in the United States is approximately 2 years; however, a profitable breakeven can be achieved only after 2.1 years of PRRSV status 4 maintenance [13,14]. Therefore, the maintenance period determines the suitability of eliminating PRRSV on a specific farm. China rears >50% of sows worldwide, and the average scale of Chinese farms is relatively large, with relatively large sow inventory; this increases pig density, along with the frequency of transportation both within and outside the farm [34]. Hence, before a PRRSV elimination project is initiated, a comprehensive assessment of the pig farm should be performed. This includes the assessment of the farm facility, biosecurity management, and major PRRSV risk factors. The PRRS risk factors include the number of accesses, movements of pigs, days with temperatures of 4–10 °C, density of farms, location of farms in sloping terrains, and location in an area with trees [35,36,37].

In the current study, the farm JS had the ideal terrain, weather conditions, and tree coverage for the elimination process; however, the risk factors included its location at a distance of <2 km from the main road and its lack of an air filtration system. Nevertheless, the exterior of its wall was obstructed by trees taller than the barns, and the exhaust of the barns faced the main road, reducing the possible negative effects of the risk factors. Moreover, its scale was moderate, leading to few operations and transportations occurring inside and outside the farm. The farm also had dedicated vehicles for piglet transport, enabling better vehicle management. However, the assessment had some limitations: (1) We evaluated JS’s surroundings at a single timepoint; in other words, we did not track the activities at the neighboring pig farms over an extended period. (2) We did not monitor the pig transport frequency on the main road. (3) The duration of PRRSV status maintenance at JS could not be quantified.

Our breeding group encompassed two operational PRRSV-positive pig farms (SG and FK) and one pig farm establishing a new group (JS). Initially, three alternative PRRSV elimination plans were proposed: (1) Procuring PRRSV-naïve replacement gilts from external sources to populate the group. (2) Introducing parity 2+ (P2+) sows from two breeding farms simultaneously and subsequently closing the herd. (3) Performing LCH after introducing weaned gilts from SG or FK. Repopulation with PRRSV-naïve gilts purchased from outside leads to a relatively high success rate of PRRSV elimination, with a short elimination period [15]. However, the genes of the group do not remain intact, and the process requires a substantial one-time investment. Eliminating PRRSV by introducing P2+ sows from SG and FK farms simultaneously before closing the herd has major advantages because most of the introduced P2+ sows have a history of PRRSV exposure, making the use of homogenized infection unnecessary [38,39]. Stability can be achieved by only providing a sufficient herd closure period and weaning piglets outside the site. However, we could not use this method because the two breeding farms were relatively small, both with a total inventory of only 800 sows, whereas the new group planned to introduce 1500 sows. As such, it would have been impossible to provide a sufficient number of P2+ sows from the two breeding farms in the short term. Moreover, the higher the frequency of sow round-trip transport, the higher is the possibility of PRRSV introduction [35,40]. Simultaneously, providing a large amount of P2+ sows can impact the overall parity structure, reducing production performance [41]. The increase in the proportion of gilts leads to an increase in PRRSV infection pressure and affects the sow herd’s reproductive performance [42,43]. Single-source introduction of weaned gilts from the group can not only preserve genetic integrity but also prevent any adverse effects on the supplier farms’ overall production performance. Therefore, we implemented the third proposed PRRSV elimination plan ultimately.

TTS is associated with PRRSV strain diversity within the herd [44]. Thus, we introduced weaned replacement gilts from one farm rather than introducing them concurrently from two PRRSV-positive unstable farms. In addition, PRRSV strain diversity in the two supplier farms was assessed. SG was eventually selected as the source farm because its resident PRRSV diversity was more homogeneous than that in FK. During the LVI process, PRRSV strain homogeneity simplifies the selection of the acclimated strain. The earlier the piglets are infected with PRRSV, the higher is the mortality and culling rate [8]. To reduce the mortality rate of introduced PRRSV-positive weaned replacement gilts during the growing period, the preweaning PRRSV infection rates of the two supplier farms were assessed systematically; it involved the collection and testing of three consecutive PF and weaning serum sample batches. Older sows have a shorter viremia duration and higher probability of birthing PRRSV-negative piglets because they may have been exposed to PRRSV more frequently [38]. Thus, for the three batches of introduced weaned gilts, the farrowing rooms were classified by parity 1 (P1) and P2+ before sow transfer, and P1 and P2+ sows were placed in these rooms accordingly. The piglets of P2+ sows were weaned first, followed by those from P1 gilts. The weaned gilts introduced to JS were only from P2+ farrowing rooms. Moreover, all weaned replacement gilts were administered antibiotics in the farrowing room before transfer to JS. However, we did not assess prevalent strains in the supplier farms through whole-genome sequencing. Prevalent PRRSV strains are relatively complex because of the presence of recombinant strains [45]. In PRRSV strains, ORF5 can be highly similar, but it only represents <5% of the whole genome; however, other genes may differ substantially [46]. This may result in a situation where if PRRSV elimination is delayed, using sequence alignment to confirm whether it was caused by a new strain’s introduction or the original strain’s circulation within the population can be difficult.

To minimize PRRSV diversity in the repopulation herd, we did not perform the herd’s MLV immunization after introduction. This may lead to a considerable increase in the mortality in the field virus circulation herd [4,47]. We attempted to reduce the number of effective contacts between infected and susceptible piglets by strengthening the biomanagement principles, decelerate PRRSV transmission within the population, and delay the whole-herd infection timepoint to reduce mortality [48]. The following specific measures were used: (1) Increasing the average rearing area to 0.44–0.50 m^2^ per weaned pig. (2) Forbidding cross-unit and cross-pen rearing from introduction to LVI. (3) Providing anti-inflammatory drugs to feverish piglets promptly, and ensuring that each pig is injected with a new needle.

The status of Batch 1 weaned gilts from PRRSV undetectable (at 3 weeks of age) to PRRSV-positive on PCR for the entire pen over 8 weeks. This outcome can serve as a reference and be used to calculate the return on investment of vaccine immunization for weaned piglets from PRRSV-positive breeding herds. Considering Batch 1 as an example, if MLV immunization is performed at 3 weeks of age and the vaccine becomes effective after 3–4 weeks [49], it could protect the uninfected pigs in 11 of 21 pens at 7 weeks of age. Similarly, if MLV immunization is conducted at 5 weeks of age and 6 of 21 pens were positive at this timepoint, then active immunity established by the vaccine may protect only the six pens at 9 weeks of age (i.e., 4 weeks later). The protection rate afforded by MLV immunization, as well as the costs of weaning piglets, vaccine, and feed, can be used to calculate the corresponding return on investment with regard to the immunization timepoint. However, the efficiency of this process may be limited when the number of piglets is relatively small (500–700 pigs per batch) and the transmission rate among different strains varies. Meanwhile, implementing cross-pen rearing and expanding the rearing space in field practices are challenging.

Compared with Batch 1, Batch 2 had a higher positivity rate at the beginning of monitoring (at 3 weeks of age), with all pens achieving positive status earlier (at 7 weeks of age). We found that with early infection, partial suckling piglets were infected with PRRSV earlier, leading to the whole batch achieving the positive status in the nursery phase. At this stage, the pigs’ passive immunity was diminished, whereas their active immunity had not been established [30]. In addition, the piglets were too young to endure a long disease course, resulting in an increased mortality or a decreased FCR [50]. This is also a reason that the mortality rate of piglets within 3–5 weeks after weaning was significantly higher when the breeding herd was at PRRSV status 1 than at status 2 [51].

We also found that several PRRSV-positive pens on PCR temporarily became negative after 2 weeks and then became positive again after another 2 weeks. This might be related to the short sample collection time; during sample collection, some piglets may have developed a fever and become reluctant to move, causing them not to want to chew the rope [27]. Another reason might be related to PRRSV infection dynamics. OF samples tested positive 1–2 days after infection and stopped shedding PRRSV after 6–7 weeks [52]. Clinical symptoms, such as fever and reluctance to chew, usually occur within 2–3 days after infection, followed by recovery in the next 14–21 days [53]. On the basis of the PRRSV infection dynamics parameters, the first set of positive results on PCR might have occurred right at infection onset in the herd without any obvious clinical symptoms. The second set of temporarily negative samples might have been collected when obvious clinical symptoms appeared, and the sick piglets did not chew the ropes. The third set of positive results might have been collected from piglets that had recovered from clinical signs but were shedding the virus. The specific reasons for these results may be verified through the collection and testing of blood samples. However, collecting blood from the entire herd incurs a high labor cost and may simultaneously accelerate PRRSV transmission within the herd.

According to compartmental models, a population may be allocated to label-bearing compartments, between which individuals can transition. The label sequence typically indicates the flow patterns among the compartments (e.g., “SEIS” indicating susceptible, exposed, infectious, and susceptible) [54]. Pigs in SIES batches undergo periodic rotation from the first to the last susceptible compartment. If a herd has both infectious and susceptible subpopulations simultaneously because of diverse exposure timepoints, PRRSV can remain in circulation within the herd. As such, we implemented homogenized infection even though most pigs were infected previously. Moreover, the clinical protective effect resulting from two consecutive infections is superior to that from a single infection [26].

Here, we employed LVI for homogenized infection, primarily because LVI has shorter mean TTS than MLV (mean TTS = 25 weeks vs. 32 weeks) [20]. In a 2024 study, median TTS of LVI was prolonged to 38 weeks [7]. In the current study, the TTS of LVI was 33 weeks. However, because no new piglets were born within 33 post-LVI weeks, classifying the PRRSV herd status according to AASV’s current assessment principles was unfeasible. Thus, the actual TTS might have been <33 weeks; nevertheless, the result fulfilled the expectations of the farm owner: Batch 1 of 55-kg gilts, which were PRRSV-naïve and ready to use and sell.

When infected by field PRRSV, mortality and ADWG improve better in the older pigs than in the younger ones [55]. Therefore, we did not apply LVI immediately on weaned gilts arriving at JS. The increased mortality rate during gilt development reduced the number of productive sows; the resulting decrease in ADWG delayed mating. In addition, we considered providing a ≥12-week cooldown period after LVI and before mating to ensure that the sows were no longer shedding the virus at mating and subsequently affecting the total piglet number per litter [56,57]. According to the two main timeline impact factors, LVI was implemented at 16 and 10 weeks of age in Batches 1 and 3, respectively.

We also found that herds with 10–16-week-old PRRSV-positive pigs stopped shedding PRRSV 12 weeks after LVI. These data can aid in isolating and determining acclimation time after the external introduction of PRRSV-positive replacement gilts. Notably, the virus shedding time is associated with herd age and PRRSV virulence [58].

According to AASV’s PRRSV classification principles, we collected PF and serum from weaning piglets and tested them to evaluate the breeding herd’s PRRSV status. Serum from perinatal sows and TTF were included in the study, mainly to detect sows and litters with potential PRRSV-positive as early as possible. In case of these sows and litters were then transferred and culled promptly through T&R. However, no PRRSV-positive pigs or litters on PCR were identified 33 weeks after LVI, indicating that the herd had attained a positive stable status 2. By continuously monitoring sow antibodies, we discovered that even when the breeding herd reached a positive stable status 2, some sows exhibited antibodies on ELISA. Under low PRRSV prevalence, passive immunity conferred by maternal antibodies might delay viremia onset, and these maternal antibodies often become negative around 8 weeks of age [30]. Thus, we performed ELISA in 8-week-old piglets to further verify the PRRSV infection and exposure status of sow herds and piglets. The results indicated that a few 8-week-old piglets in Batch 1 remained positive on ELISA but became negative by 10 weeks of age, whereas all 8-week-old piglets in the other three batches remained negative on ELISA. This result, in combination with the PCR results of four consecutive batches in the farrowing room, further confirmed that the breeding herd had reached a positive stable status 2.

When a breeding herd attains the positive stable status 2, the 55-kg PRRSV-naïve gilts within the herd can be sold successfully. If a farm owner desires to further achieve a negative status 4, they must accomplish replacement according to the annual gilt replacement plan. For instance, if the annual replacement rate is 50%, then it will take only 2 years to complete the full herd replacement and status 4 achievement. To expedite the elimination process and concurrently mitigate the related impact on normal production operations, we employed T&R. After each sow batch was weaned, antibody-positive sows were screened out through ELISA, and self-breeding PRRSV-naïve gilts were used for replacement. The PRRSV-negative sows on ELISA were retained to minimize losses. Our T&R method for the weaned sow herd significantly differed from that applied previously [17,59]. The advantages of this method are as follows: (1) Number of culled sows is minimized to the greatest extent possible. (2) Organizing considerable manpower in a short period to collect and test samples for an entire herd is hard, but it can increase the frequency of cross-contamination within the herd. (3) Each batch’s outputs are guaranteed to the maximum extent during the T&R period. (4) Selling PRRSV-naïve gilts not used for the whole sow herd’s culling and replacement enhances the farm’s revenue.

If a farm’s system runs its own PRRSV-naïve breeding farms, offsite breeding can be conducted in advance. After the breeding herd attains positive stable status 2, a sufficient number of pregnant, ready-to-mate PRRSV-naïve gilts can be introduced simultaneously to restore full production promptly. This is also an alternative approach currently employed by groups during PRRSV elimination through LCH. This method can minimize opportunity losses during self-breeding gilts on site (i.e., minimize loss of weaned pigs). In the current study, after all seven T&R and rollover batches, we performed two rounds of ELISA testing by using randomized samples from the sow herd, and all samples tested negative, demonstrating that the farm had successfully attained the PRRSV-negative status 4. Finally, PRRSV-naïve gilts sold by JS received positive customer reviews.

## 5. Conclusions

Here, we developed and executed systematic, step-by-step PRRSV elimination protocols. They encompassed pre-evaluation of the PRRSV elimination capacity of new repopulation farm, assessment of PRRSV status and strain diversity of the two supplier farms, analysis of the natural infection dynamics of PRRSV within the population, assessment of parameters for LVI material preparation and implementation, and detailed programs for monitoring changes from LVI to PRRSV-negative status 4 achievement in the herd. PRRSV elimination was initiated with the introduction of group internal weaned PRRSV-positive gilts, which maintained genetic integrity, reduced the costs of purchasing gilts from external sources, and further mitigated the operational complexity of introducing sows before LCH application.

## Figures and Tables

**Table 1:** Monitoring strategies for SG and FK.

Sample Type	Pig Age (Days)	No. of Samples	Pooling Strategies
PF	3–5	3–6	Pooling of samples from every 30–50 L
Serum	21–25	30	Pooling of five samples
OF	42–46	6	-

**Table 2:** Strategies for sampling after homogenized infection via LVI.

Sample Type	No. of Samples	ELISA or PCR	Sampling Timepoint	Objective
OF	All pens	PCR	12–24 h after challenge	Evaluating intervention efficiency
Serum	≥30	ELISA	4 weeks after challenge	Assessing infection rate
OF	All pens	PCR	Every 4 weeks until all pens became qPCR negative	Verifying shedding termination in the herd
Serum	≥30	ELISA	Every 3 weeks until T&R end (excluding some exceptions)	Monitoring exposure status dynamics

**Table 3:** Strategies for PRRSV monitoring the achievement of PRRSV herd status 2 from status 1.

Sample Type	Piglet Age (Days)	No. of Samples	Pooling Strategy	PCR or ELISA
TTF	Stillborn	2	20–100 tongue tips per sample	PCR
PF	3–5	7–8	30–50 L per sample	PCR
Serum	21–25	120–240	5 pigs per pool	PCR
Serum	56	120	-	ELISA
Serum	Perinatal sows	60	5 sows per pool	PCR

**Table 4:** Strategies for PRRSV monitoring the achievement of PRRSV herd status 4 from status 2 and then status 3.

Herd	Sample Type	No. of Samples	ELISA or PCR	Sampling Timepoint	Objective
Weaned sows	Serum	All weaned sows in a batch	ELISA	After weaning	Screening antibody positivity in sows for replacement with naïve gilts
Sentinel gilts	Serum	30	ELISA	0, 30, and 60 days after commingling with sow herd	Ensuring achievement of herd status 3, according to AASV PRRS classification
Sows	Serum	60	ELISA	Accomplishment of whole-herd R&T; randomly selection of 60 sows for ELISA testing 30 and 90 days after T&R and rollover	Verifying achievement of herd status 4

**Table 5:** PRRSV monitoring results at FK.

Batch No.	Sample Type	No. of Positive qPCR Results	No. of qPCR Results	Percentage of Positive qPCR Results	PRRSV Lineage
20220514	PF	0	4	0%	-
	Weaning serum	5	6	83%	5
	Nursery serum	6	6	100%	5
20220612	PF	0	6	0%	-
	Weaning serum	7	12	58%	5
	Nursery serum	6	6	100%	3, 8.7
20220626	PF	0	4	0%	-
	Weaning serum	3	6	50%	8.7
	Nursery serum	6	6	100%	5

**Table 6:** PRRSV monitoring results at SG.

Batch No.	Sample Type	No. of Positive qPCR Results	No. of qPCR Results	Percentage of Positive qPCR Results	PRRSV Lineage
20220514	PF	0	3	0%	-
	Weaning serum	6	6	100%	8.7
	Nursery serum	6	6	100%	5
20220612	PF	1	6	17%	FALSE
	Weaning serum	3	12	25%	8.7
	Nursery serum	5	6	83%	5
20220626	PF	0	6	0%	-
	Weaning serum	1	6	17%	8.7
	Nursery serum	6	6	100%	8.7

**Table 7:** PRRSV N protein positivity rate over 4–72 weeks after challenge.

	4 WPC ^1^	16 WPC	19 WPC	22 WPC	25 WPC	28 WPC	31 WPC	44 WPC	57 WPC	60 WPC	63 WPC	66 WPC	69 WPC	72 WPC
No. of positive samples	366	29	43	53	51	49	45	31	10	12	10	7	8	4
No. of samples	369	30	60	60	61	61	61	120	90	119	158	84	100	122
Percentage of positive samples	99% ^a^	97% ^a^	72% ^b^	88% ^b^	84% ^b^	80% ^b^	74% ^b^	26% ^c^	11.1% ^d^	10.1% ^d^	6.3% ^d^	8.3% ^d^	8.0% ^d^	3.3% ^e^

^1^ WPC, weeks after challenge; Groups sharing the same letter imply no statistically significant difference in positive rates, whereas groups with different letters suggest significant differences and *p*-value was less than 0.00055.

**Table 8:** qPCR results in suckling piglets from four consecutive batches.

Batch	Birthdate	Sample Type	No. of Positive qPCR Results	No. of qPCR Results	Percentage of Positive qPCR Results
Batch 1	4 July 2023	TTF	0	2	0%
		PF	0	7	0%
		Serum	0	48	0%
Batch 2	25 July 2023	TTF	0	2	0%
		PF	0	7	0%
		Serum	0	24	0%
Batch 3	15 August 2023	TTF	0	2	0%
		PF	0	7	0%
		Serum	0	24	0%
Batch 4	5 September 2023	TTF	0	2	0%
		PF	0	8	0%
		Serum	-	-	-

**Table 9:** qPCR results in perinatal sows from seven consecutive batches.

Batch	No. of Positive qPCR Results	No. of qPCR Results	Percentage of Positive qPCR Results
Batch 1	0	6	0%
Batch 2	0	12	0%
Batch 3	0	12	0%
Batch 4	0	12	0%
Batch 5	0	12	0%
Batch 6	0	12	0%
Batch 7	0	12	0%

**Table 10:** ELISA results in 8-week-old piglets from four consecutive batches.

Batch	Mean S/P Value	No. of Positive Samples	No. of Samples	Percentage of Positive Samples
Batch 1	0.11	1	120	1.67%
Batch 2	−0.14	0	120	0%
Batch 3	−0.12	0	120	0%
Batch 4	0.03	0	120	0%

**Table 11:** ELISA results in 30 sentinel gilts at 0, 30, and 60 days after introduction.

	0 DPI ^1^	30 DPI	60 DPI
Mean S/P	−0.08	−0.08	−0.07
CV	0.11	0.07	0.11

^1^ DPI, days after introduction.

**Table 12:** ELISA results in weaned sows from seven consecutive batches.

Batch	Mean S/P Value	No. of Positive Samples	No. of Samples	Percentage of Positive Samples
Batch 1	0.077	11	90	12.2% ^a^
Batch 2	0.003	12	119	10.1% ^a^
Batch 3	0.015	10	108	9.3% ^ab^
Batch 4	0.023	10	158	6.3% ^ab^
Batch 5	0.055	7	84	8.3% ^ab^
Batch 6	0.013	8	100	8.0% ^ab^
Batch 7	−0.013	4	122	3.3% ^b^

Groups sharing the same letter imply no statistically significant difference in positive rates, whereas groups with different letters suggest significant differences and *p*-value was less than 0.0024.

**Table 13:** ELISA results in productive sows at 30 and 90 days after T&R and rollover completion.

	30 Days After T&R and Rollover	90 Days After T&R and Rollover
Mean S/P value	−0.01	0.02
CV	0.14	0.04

## Data Availability

The original contributions presented in this study are included in the article/supplementary material. Further inquiries can be directed to the corresponding author(s).

## Supplementary Materials

The following supporting information can be downloaded at: https://www.mdpi.com/article/10.3390/vetsci12101012/s1, Table S1: ORF5 sequence list.

## Abbreviations

The following abbreviations are used in this manuscript:

PRRS	Porcine reproductive and respiratory syndrome
PRRSV	Porcine reproductive and respiratory syndrome virus
LVI	Live virus inoculation
MLV	Modified live vaccine
LCH	Load close and homogenize
T&R	Test and removal
ELISA	Enzyme-linked immunosorbent assay
PCR	Polymerase chain reaction
TTF	Tongue tip fluids
PF	Processing fluids
OF	Oral fluids
TTS	Time to stability
TTBP	Time to baseline productivity
TL	Total loss
AASV	American Association of Swine Veterinarians
ADWG	Average daily weight gain
ORF	Open reading frame
DPTRA	Days post T&R accomplished
DPI	Days post introduction
WPC	Weeks post challenged
