# Supplementary material for: Combining Load–Close–Homogenize with Testing, Removal, and Rollover Strategies to Repopulate PRRSV Elimination Breeding Herds Using PRRSV-Positive Weaned Gilts
Source: Vet Sci. 2025 Oct 20;12(10):1012. doi: 10.3390/vetsci12101012 (PMC12567787; doi:10.3390/vetsci12101012)
Supplement: Supplementary file 1 [file vetsci-12-01012-s001.zip › vetsci-3883167-supplementary material.pdf]

Supplementary Table S1: ORF5 sequence list.
FK-1
TGTTGGAGGAGTGCTTGACCGCATGCTGTTGCTCGCGATTGCTTTTTTTGTGGTGTATCGTGCCG
TTCTATCTTGCTGTGCTCGTCAACGCCAGCAACAACAACAGCTCTCATATTCAGTTGATTTATAA
CTTAACGTTATGTGAGCTGAATGGCACAGATTGGCTGGTACAAAAATTTGACTGGGCAGTGGAG
ACTTTTGTCATTTTCCCCGTGTTGACTCACATCGTTTCCTACGGGGCACTCACCACCAGCCATTTC
CTTGACACAGTTGGTTTGGCCACTGTGTCCACCGCCGGATATTATCACGGGCGGTATGTCTTGA
GTAGCATTTACGCAGTCTGTGCTCTGGCTGCGCTGATTTGCTTTGTCATTAGGCTTGCGAAGAAC
TGCATGTCCTGGCGCTACTCTTGTACCAGATATACCAATTTCCTTCTGGACACTAAGGGCAGACT
CTATCGTTGGCGGTCGCCCGTCATTGTGGAGAAAGGGGGTAAGGTTGAGGTCGAAGGTCACCT
GATCGACCTCAAGAGAGTTGTGCTTGATGGTTCCGCGGCAACCCCTTTAACCAGAGTTTCAGCG
GAACGATGGGGTCGTCCCTAG
FK-2
GCTGTGCTCGTCACGCCAGCAACACAACAGCTCTCATATTCAGTTGATTTATAACTTAACGTTAT
GTGAGCTGAATGGCACAGATTGGCTGGCACAAAAATTTGACTGGGCAGTGGAGACTTTTGTCAT
CTTCCCCGTGTTGACTCACATCGTTTCCTATGGGGCACTCACCACCAGCCATTTCCTTGACACAG
TTGGTTTGGCCACTGTGTCCACCGCCGGATATTATCACGGGCGGTATGTCTTGAGTAGCATTTAC
GCAGTCTGTGCTCTGGCTGCGCTGATTTGCTTTGTCATTAGGCTTGCGAAGAACTGCATGTCCTG
GCGCTACTCTTGTACCAGATATACCA
JS-1
ATGTTGGGGAAGTGCTTGACCGCATGCTGTTGCTCGCGATTGCTTTTTTTGTGGTGTATCGTGCC
GTTCTATTTTGCTGTGCTCGTCAACGCCGGCAACAACAACAGCTCTCATATTCAGTTGATTTATA
ATTTGACGTTATGTGAGCTGAATGGCACAGATTGGCTGGCAGAAAATTTTGACTGGGCAGTGGA
GACTTTTGTCATCTTCCCCGTGTTGACCCACATTGTTTCCTATGGGGCACTCACCACCAGCCATTT
CCTCGACACAGTTGGTCTGGCCACTGTGTCTACCGCCGGATATTATCACGGGCGGTATGTCTTG
AGTAGCATTTACGCGGTCTGTACTCTGGCTGCGCTGATCTGTTTTGTCATTAGGCTTGCGAAGAA
CTGCATGTCTTGGCGCTACTCTTGTACCAGATATACCAACTTCCTTCTTGACACTAAGGGCAAAC
TCTATCGTTGGCGGTCGCCCGTTATTGTGGAGAAAGGGGGTAAGGTTGAGGTCGAAGGTCACCT
GATCGACCTCAAGAGAGTTGTGCTTGATGGTTCCGCGGCAACCCCTTTAACCAGAGTTTCAGCG
GAACGATGGGGTCGTCCTTAG
JS-2
TTTGCCATCCTACTGGCAATTTGAATGTTCAAGTATGTTGGGGAAGTGCTTGACCGCATGCTGTT
GCTCGCGATTGCTTTTTTTGTGGTGTATCGTGCCGTTCTATTTTGCTGTGCTCGTCAACGCCAGCA
AAAACAACAGCTCTCATATTCAGTTGATTTATAATTTGACGTTATGTGAGCTGAATGGCACAGAT
TGGCTGGCAAAAAATTTTGACTGGGCAGTGGAGACTTTTGTCATCTTCCCCGTGTTGACCCACAT
TGTTTCCTATGGGGCACTCACCACCAGCCATTTCCTTGACACAGTTGGTCTGGCCACTGTGTCTA
CCGCCGGATATTATCACGGGCGGTATGTCTTGAGTAGCATTTACGCGGTCTGTACTCTGGCTGC
GCTGATCTGTTTTGTCATTAGGCTTGCGAAGAACTGCATGTCTTGGCGCTACTCGTGTACCAGAT
ATACCAACTTCCTTCTTGACACTAAGGGCAAACTCTATCGTTGGCGGTCGCCCGTTATTGTGGAG
AAAGGGGGTAAGGTTGAGGTCGAAGGTCACCTGATCGACCTCAAGAGAGTTGTGCTTGATGGT
TCCGCGGCAACCCCTTTAACCAGAGTTTCAGCGGAACGATGGGGTCGTCCCTAGACGACTTCTG
CAATGATAGCACGGCTCCACAAA
SG-1
ATGTTGGGGAAGTGCTTGACCGCATGCTGTTGCTCGCGATTGCTTTTTTTGTGGTGTATCGTGCC

GTTCTATTTTGCTGTGCTCGTCAACGCCAGCAACAACAACAGCTCTCATATTCAGTTGATTTATAA
TTTGACGTTATGTGAGCTGAATGGCACAGATTGGCTGGCAGAAAAATTTGACTGGGCAGTGGAG
ACTTTTGTCATCTTCCCCGTGTTGACCCACATTGTTTCCTATGGGGCACTCACCACCAGCCATTTC
CTCGACACAGTTGGTCTGGCCACTGTGTCTACCGCCGGATATTATCACGAGCGGTATGTCTTGA
GTAGCATTTACGCGGTCTGTACTCTGGCTGCGCTGATCTGTTTTGTCATTAGGCTTGCGAAGAAC
TGCATGTCTTGGCGCTACTCTTGTACCAGATATACCAACTTCCTTCTTGACACTAAGGGCAAACT
CTATCGTTGGCGGTCGCCCGTTATTGTGGAGAAAGGGGGTAAGGTTGAGGTCGAAGGTCACCT
GATCGACCTCAAGAGAGTTGTGCTTGATGGTTCCGCGGCAACCCCTTTAACCAGAGTTTCAGCG
GAACGATGGGGTCGTCCTTAG
SG-2
ATGTTGGGGAAGTGCTTGACCGCATGCTGTTGCTCGCGATTGCTTTTTTTGTGGTGTATCGTGCC
GTTCTATTTTGCTGTGCTCGTCAACGCCAGCAACAACAACAGCTCTCATATTCAGTTGATTTATAA
TTTGACGTTATGTGAGCTGAATGGCACAGATTGGCTGGCAGAAATATTTGACTGGGCAGTGGAG
ACTTTTGTCATCTTCCCCGTGTTGACCCACATTGTTTCCTATGGGGCACTCACCACCAGCCATTTC
CTCGACACAGCTGGTCTGGCCACTGTGTCTACCCCCGGATATTATCACGGACGGTATGTCTTGA
GTAGCATTTACGCGGTCTGTACTCTGGCTGCGCTGATCTGTTTTGTCATTAGGCTTGCGAAGAAC
TGCATGTCTTGGCGCTACTCTTGTACCAGATATACCAACTTCCTTCTTGACACTAAGGGCAAACT
CTATCGTTGGCGGTCGCCCGTTATTGTGGAGAAAGGGGGTAAGGTTGAGGTCGAAGGTCACCT
GATCGACCTCAAGAGAGTTGTGCTTGATGGTTCCGCGGCAACCTCTTTAACCAGAGTTTCAGCG
GAACGATGGGGTCGTCCTTAG
SG-3
ATGTTGGGGAAGTGCTTGACCGCATGCTGTTGCTCGCGATTGCTTTTTTTGTGGTGTATCGTGCC
GTTCTATTTTGCTGTGCTCGTCAACGCCAGCAACAACACCAGCTCTCATATTCAGTTGATTTATAA
TTTGACGTTATGTGAGCTGAATGGCACAGATTGGCTGGCAAAAAAATTTGACTGGGCAGTGGAG
ACTTTTGTCATCTTCCCCGTGTTGACCCACATTGTTTCCTATGGGGCACTCACCACCAGCCATTTC
CTTGACACAGTTGGTCTGGCCACTGTGTCTACCGCCGGATATTATCACGGGCGGTATGTCTTGA
GTAGCATTTACGCGGTCTGTACTCTGGCTGCGCTGGTCTGTTTTGTCATTAGGCTTGCGAAGAAC
TGCATGTCTTGGCGCTACTCTTGTACCAGATATACCAACTTCCTTCTTGACACTAAGGGCAAACT
CTATCGTTGGCGGTCGCCCGTTATTGTGGAGAAAGGGGGTAAGGTTGAGGTCGAAGGTCACCT
GATCGACCTCAAGAGAGTTGTGCTTGATGGTTCCGCGGCAACCCCTTTAACCAGAGTTTCAGCG
GAACGATGGGGTCGTCCCTAG
SG-4
ATGTTGGGGAAGTGCTTGACCGCATGCTGTTGCTCGCGATTGCTTTTTTTGTGGTGTATCGTGCC
GTTCTATCTTGCTGTGCTCGTCAACGCCGGCAACAACAACAGCTCTCATATTCAGTCGATTTATA
ATTTAACGTTATGTGAGCTAAACGGCACAGATTGGCTGGCCAGGAAATTTGACTGGGCAGTGGA
GACTTTTGTCATCTTCCCCGTGTTGACCCACATTGTTTCCTATGGGGCACTCACCACCAGCCATTT
CCTTGACACAGTTGGTCTGGCCACTGTGTCTACCGCCGGATATTATCACAGGCGGTATGTCTTGA
GTAGCATTTACGCGGTCTGTGCTCTGGCTGCGCTGATCTGCTTTGTCATTAGGCTTGCGAAGAAC
TGCATGTCTTGGCGCTACTCTTGTACCAGATATACCAACTTCCTTCTCGACACTAAGGGCAAACT
CTATCGTTGGCGGTCGCCCGTCATCGTGGAGAAAGAGGGTAAGGTTGAGGTCGAAGGTCACCT
GATCGACCTCAAGAGAGTTGTGCTTGATGGTTCCGCGGCAACCCCTTTAACCAGAGTTTCAGCG
GAACGATGGGGTCGTCCCTAG

Lineage 8.7_JXA1_GenBank_MT163314.1

Lineage 1.8_NADC30_GenBank_JN654459.1
Lineage 1.5_NADC34_GenBank_MN648450.1
Lineage 3_QYYZ_GenBank_JQ308798.1
Lineage 5_RespPRRS MLV_GenBank_AF066183.4
Lineage 5_VR2332_GenBank_EF536003.1
